# Implementation and acceptability of a community based intervention for cervical cancer screening amongst women of reproductive age in Kiambu County, Kenya

**DOI:** 10.1186/s13104-026-07710-z

**Published:** 2026-03-15

**Authors:** Redempta Mutisya, Eliphas Gitonga, Molly Mukii Maundu, Rosebella Iseme-Ondiek

**Affiliations:** 1 Department of Environmental and Occupational Health, School of Health Sciences, Kenyatta University, P. O. Box 438-44-00100, Nairobi, Kenya; 2 Department of Pathology and Laboratory Medicine, Kiambu Level 5 Hospital, Kiambu County, P. O. Box 39-00900, Kiambu, Kenya; 3 Department of Population Health, Medical College, Aga Khan University, P. O. Box 30270-00100, Nairobi, Kenya

**Keywords:** Cervical cancer screening, Self-sampling, Short message service (SMS), Screening uptake, Feasibility

## Abstract

**Objective:**

In Kenya, cervical cancer is the top cause of cancer deaths and second most prevalent cancer among women aged 15–44. Despite the existence of screening programs, only 13.8% of women participate, with nearly 50% diagnosed late. Lack of knowledge and embarrassment is linked to low uptake. Post-intervention, uptake of screening and acceptability of SMS prompts and self-sampling were evaluated.

**Methods:**

Women (18+ years), who had never screened, were randomly chosen from a pre-existing database. They received either usual care (notification of availability of screening services at public health facility), text education, or interactive in-person sessions with self-sample training.

**Results:**

85 women received health education and self-sample kits, 55 received SMS prompts, and 36 received usual care. Screening rates were highest in the self-sample group (53%). 78% of self-sample participants recommended the Evalyn brush. Only about 52% of SMS recipients found the messages useful, and just 31% were open to receiving more educational messages. Community health workers were instrumental in the implementation of self-sample collection.

**Conclusions:**

Community-based strategies leveraging existing resources and incorporating education and self-sampling demonstrate feasibility and potential acceptability, particularly in culturally rooted areas with geographical access barriers. Careful design of messaging strategies for behavior change remains crucial.

*Trial registration* The trial was approved for registration with the Pan African Clinical TrialRegistry on 21/09/2023 (Clinical Trial No. PACTR202309878831811). https://pactr.samrc.ac.za/TrialDisplay.aspx?TrialID=25484.

## Background

Cervical cancer is a major global health issue, ranking fourth in incidence and cancer-related mortality, accounting for 13.1% of all female cancers and causing approximately 350,000 deaths in 2022 [1]. Notably, 80% of cases and 90% of deaths occur in low and middle-income countries (LMICs). Africa has the highest incidence rates and Eastern Africa records the highest mortality rates at 30.0 per 100,000 [2]. In Kenya, cervical cancer is the leading cause of cancer-related deaths and the second most common cancer among women aged 15 to 44, with around 4800 diagnoses and 2400 deaths each year [3].

Efforts to control and prevent cervical cancer include human papillomavirus (HPV) vaccination, screening, treatment of precancerous lesions, and palliative care [1, 4]. Cervical cancer screening is vital for early detection and effective intervention, yet uptake remains low, even with free services available. sub-Saharan Africa (SSA) has the lowest prevalence of cervical cancer screening uptake [5, 6]. In Kenya, only 13.8% of reproductive-age women have participated in screening, with rates dropping below 11% in rural areas and nearly 50% presenting with late-stage disease [7–9]. The latter is attributed to a combination of individual (lack of awareness, knowledge, fear, beliefs, financial constraints), community (stigma) and health system factors (limited screening centres in rural areas, complex referral pathways, inconsistent availability of tests and trained staff) with lack of awareness, distance to the nearest health facility offering screening and beliefs around the screening process presenting as major impediments [10–12]. It has been projected that if current trends continue, by 2030, 98% of cervical cancer deaths will occur in developing countries [13].

High risk HPV screening (HR-HPV) has been shown to reduce cervical cancer incidence in countries across the world [14, 15]. The latter test shows whether high-risk HPV types (, i.e. any one or more of fourteen HPV strains linked to the occurrence of several types of cancer – HPV 31, 33, 35, 39, 45, 51, 52, 56, 58, 59, 66, 68 as well as 16 and 18, two important strains which cause most HPV related cancer) were found in cervical cells. The current national guidelines on cervical cancer screening recommend HR-HPV screening as a primary method for women > 30years [16]. However, its adoption has been slow. This is largely explained by Kenya’s reliance on an opportunistic screening approach, in which services are offered to individuals who present at health facilities and either request screening or are referred for it, rather than through systematic population-based programs or proactive demand generation via community- or facility-based information, education, and communication (IEC) campaigns (such as short message service prompts or the dissemination of educational materials). This study aimed to pilot a multi-modal intervention to increase HR-HPV screening uptake by offering women convenient self-sampling, health education, and enhanced partner support. Due to a low response rate, the study was not conducted as originally planned. This research note presents findings on the implementation and acceptability of community-based self-sampling for cervical cancer screening.

## Materials and methods

### Study design

The research utilized a randomized controlled trial design (specifically, a parallel design) and ran between April 2022–July 2023. A detailed description of the study methodology has been previously reported [7].

### Study area

Kiambu County is recognized to be amongst the top ten counties with the highest prevalence of cervical cancer within Kenya [17]. Furthermore, the rate of utilization of cervical cancer screening is noted to be low [18, 19]. A baseline assessment highlighted a fear of the screening procedure as being amongst the key barriers to seeking this service [7]. A map of Kiambu County is presented in Appendix 1.

### Target population and sample size

**Fig. 1:**
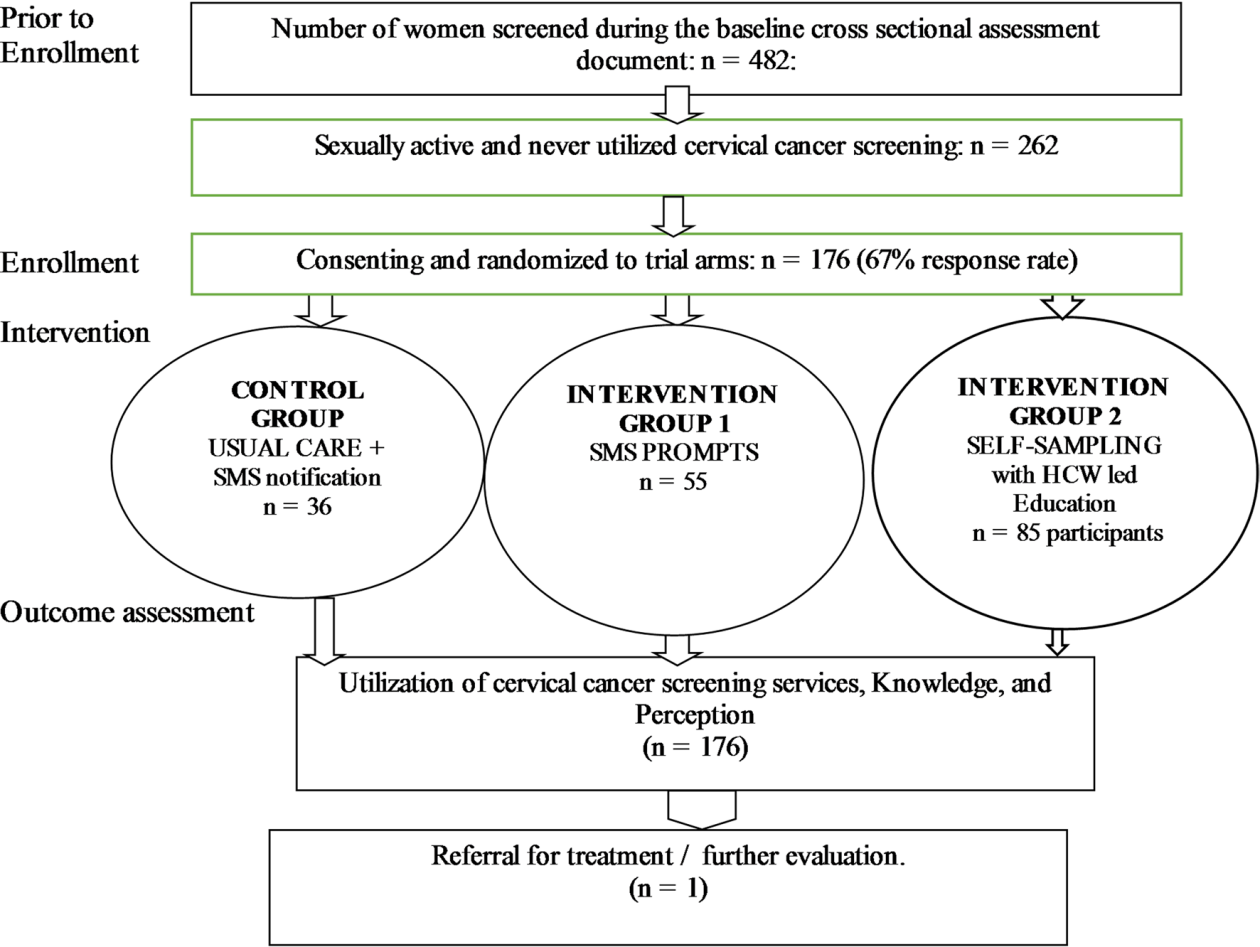
Flow diagram of the HPV self-sampling kits versus provider-initiated short message service (SMS) prompts cervical cancer screening intervention study

The target population comprised women of reproductive age (15–49 years) who had previously acknowledged that they were sexually active and had never utilised cervical cancer screening services (n = 262) [7]. The latter were followed up with a telephone call and invited to participate in the intervention study. Only 176 study participants provided informed consent (including their contacts and permission to be contacted) to participate in the intervention study. Figure 1 outlines study participant recruitment.

### Recruitment and sampling

Study participants were drawn from all four wards (Riabai, Ting’ang’a, Kiambu Township and Ndumberi) of Kiambu sub-county. Random sampling was used to select the households from which study participants were drawn using community health volunteer (CHV) household (HH) registers as well as to select one female respondent in households with more than one eligible person.

### Randomization

Randomization was undertaken using STATA version 13 (StataCorp, 2013). An unequal randomization ratio was used to assign participants to one of three groups. This approach allowed the study team to maximize public health benefit within the constraints of available funding. This approach allowed a greater proportion of participants to receive self-sampling kits and health education interventions, thereby enhancing the intervention’s reach and our ability to assess its acceptability.

### Intervention

**Fig. 2:**
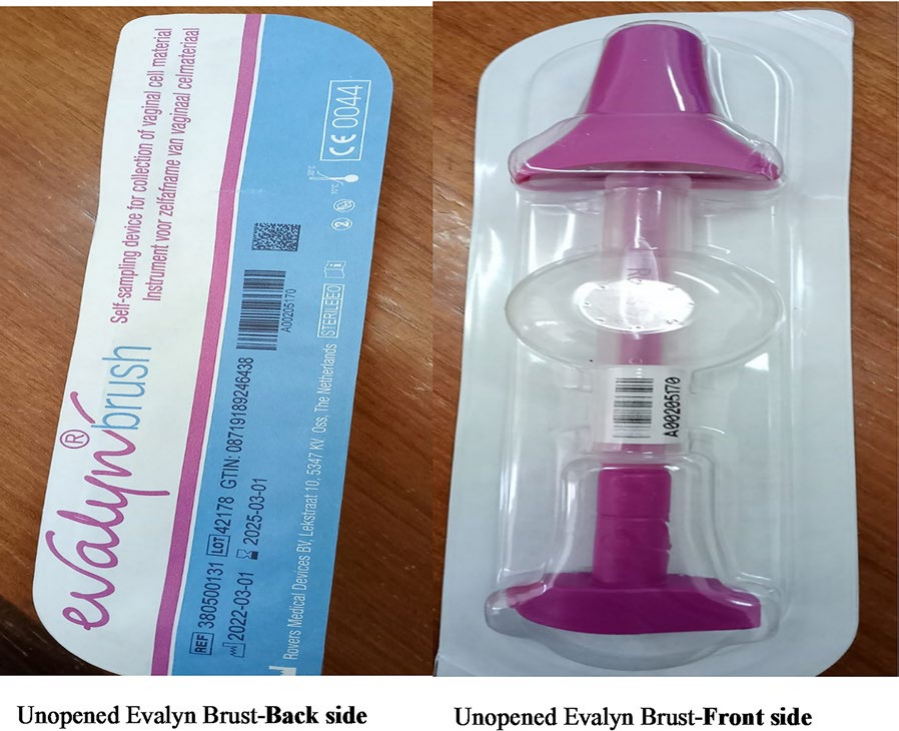
HPV self-sampling kit

Study participants were assigned to one of two intervention groups. The first group (Intervention Group 1) received health education via Ministry of Health-approved mobile text messages (SMS prompts), sent twice a week for two weeks, covering topics such as cervical cancer screening, early detection, risk factors, and accessing free services in both English and Kiswahili (Appendix 2). The second group (Intervention Group 2) (self-sample with HCW led education) attended a one-day interactive health education session led by healthcare workers (HCWs) from Kiambu Level 5 Hospital. This session covered similar topics as those covered in the SMS prompts as well as included training on using the Evalyn Brush for self-sampling as per manufacturer guidelines [20]. Participants were provided with kits (Fig. 2) and either collected samples on-site or took them home. CHVs collected the samples twice a week and transported them at ambient temperature for testing at Kiambu Level 5 Hospital (1–2-hour maximum travel time). Participants were notified via SMS when their results were ready for pickup.

### Control

Women in the control arm (usual care + SMS notification) could still access free cervical cancer screening services at Kiambu Level 5 Hospital, as part of the national screening program. The available screening methods include Visual Inspection with Acetic Acid (VIA), Visual Inspection with Lugol’s Iodine, and Pap smears. According to the guidelines, any woman who has ever had sexual intercourse is eligible for screening [21]. Currently, the government neither distributes educational materials nor sends routine nationwide SMSs with information on where to access free screening services under the national program. However, women in our sample did receive a single SMS notification regarding the availability of these services at Kiambu Level 5 Hospital.

### Laboratory analyses

Participants collected samples using the dry Evalyn self-sampling brush, placed them in zip-lock bags which were collected by CHVs and delivered to the Kiambu Level 5 laboratory on the same day as sample collection. The samples were handled as high-risk biohazards, with universal precautions followed. The laboratory used the Xpert^®^HPV assay (Cepheid) to detect high-risk HPV types by targeting the E6/E7 region of the viral DNA, including 14 hrHPV types. The test provides partial genotyping for HPV 16 and 18/45 and is validated for primary cervical screening [22]. Sample processing and reporting followed the Ministry of Health Standard Operating Procedures [16]. Results were reported as invalid, positive, or negative for HPV types 16, 18/45, and 11 other high-risk types. Women with positive results were referred for VIA screening, as per national guidelines [23].

### Data collection tools and procedures

An exit survey, conducted 6 months after the last educational session, assessed women’s knowledge of cervical cancer and perceptions of screening methods. Data were collected via an electronically administered, semi-structured questionnaire using Kobo Collect software. The survey also evaluated the acceptability of self-sampling and the demand for cervical cancer screening post-intervention. For the self-sampling group, demand was based on successful sample collection by CHVs, while for the SMS prompt and usual care groups, demand was defined as participants reporting a cervico-vaginal sample collected at a health facility.

### Data management and analysis

Data was downloaded from Kobo in an excel worksheet and imported into STATA for cleaning and analyses. To check the quality of the data, we looked for completeness, correctness of responses and skip patterns. The characteristics of the women in the study sample including acceptance of and demand for the intervention, were described using frequency counts and proportions and means and standard deviations, as appropriate. All analyses were performed using STATA version 13 (College Station, TX: StataCorp LP).

### Ethical considerations

The study received ethical approval from the Kenyatta University Ethical Review Committee (Approval No: PKU/2333/11472) and a research permit from the National Commission for Science, Technology, and Innovation (NACOSTI License No: NACOSTI/P/21/13550). Approvals were also obtained from the County offices, health facilities, and study participants (informed consent). Data security measures, including use of unique identifiers, encryption and password protection, were implemented. After the exit survey, the control group received an interactive education session. The trial is registered with the Pan African Clinical Trial Registry (Clinical Trial Number: PACTR202309878831811).

## Results

### Socio-demographic and clinical characteristics

176 out of 262 women invited, participated in the study (62% response rate). Table 1 presents the participants’ characteristics. Values shown in bold represent the most common category within each variable.

**Table 1:** Study participant sociodemographic and clinical characteristics

Demographic characteristic	Control group Usual care + SMS Notification (n = 36) Mean (± SD); n (%)	Intervention group 1 SMS prompts (n = 55)	Intervention group 2 Self-sample with HCW led education (n = 85) Mean (± SD); n (%)
Age	32 years (± 13.5 years)	Mean (± SD); n (%)	37 years (± 9.7 years)
Religion	**Protestant - 25 (69.4%)**	34 years (± 9.5 years)	**Protestant - 58 (68.2%)**
	Catholic - 11 (30.6%)	Protestant - 41 (74.5%)	Catholic - 24 (28.2%)
	Other - 0 (0%)	Catholic - 14 (25.5%)	Other - 3 (3.5%)
Occupation	**Unemployed - 16 (44.4%)**	Other - 0 (0%)	**Unemployed - 26 (30.6%)**
	Self-employed - 10 (27.8%)	Unemployed - 13 (23.6%)	Self-employed - 20 (24.4%)
	Employed - 8 (22.2%)	Self-employed - 19 (34.5%)	Employed - 17 (20.0%)
	Casual - 2 (5.6%)	Employed - 6 (10.9%)	Casual - 22 (25.9%)
Level of Education	No formal education - 0 (0%)	Casual - 17 (30.9%)	No formal education - 10 (11.8%)
	Primary - 10 (27.8%)	No formal education - 2 (3.6%)	Primary - 28 (32.9%)
	**Secondary - 20 (55.6%)**	Primary - 21 (38.2%)	**Secondary - 31 (36.5%)**
	Tertiary - 6 (16.7%)	Secondary - 29 (52.7%)	Tertiary - 16 (18.8%)
Marital Status	**Single (never married) - 18 (50%)**	Tertiary - 3 (5.5%)	**Single (never married) - 49 (57.6%)**
	Married - 18 (50%)	Single (never married) - 33 (60%)	Married - 30 (35.3%)
	Other - 0 (0%)	Married - 16 (29.1%)	Other - 6 (7.1%)
Source of payment for health services	Employer health expenditure - 0 (0%)	Other - 6 (10.9%)	Employer health expenditure - 1 (1.2%)
	Private heath expenditure - 2 (5.6%)	Employer health expenditure - 0 (0%)	Private heath expenditure - 0 (0%)
	**Out of pocket (self or family member) - 13 (36.1%)**	Out of pocket (self or family member) - 30 (54.5%)	**Out of pocket (self or family member) - 61 (71.8%)**
	NHIF - 21 (58.3%)	NHIF - 24 (43.6%)	NHIF - 23 (27.1%)
Uptake of cervical cancer screening	Yes - 8 (22.2%)	Yes - 11 (20%)	Yes - 45 (52.9%)

Among the 85 women who received in-person HCW led health education and a self-sampling kit, 52.9% (n = 45) utilized the cervical cancer screening services. Alternatively, only 20% (n = 11) of those receiving usual care alongside the SMS notification (n = 36) and 22.2% (n = 8) of those receiving SMS prompts (n = 55) took up the screening services (refer to Table 1). Additionally, the distribution of occupations varied across the three groups. Whilst the unemployed formed the largest category in all three groups, casual workers (comprising individuals hired without formal contracts paid on a day to day basis) comprised a much smaller proportion of participants randomized to the control group (n = 2; 5.6%), compared with those in intervention group 1 (n = 17; 30.9%) and intervention group 2 (n = 22; 25.9%). Notably, participants reported experiencing a range of emotions while waiting for results, including fear, anxiety, or both. Only 26.6% of participants felt calm during the waiting period (refer to Appendix 3).

### Study participant perception regarding cervical cancer screening

Of the women screened through self-sample collection (n = 45), a positive attitude was noted, with majority of women (78.2%) stating they would recommend the use of the Evalyn brush to female family, although 37.8% stated they did experience some pain/discomfort using the brush. Alternatively, though most respondents who received SMS prompts believed the messages were culturally appropriate (81.8%), informative (52.7%) and educational (61.8%), less than half (49.1%) felt the number of messages was adequate and even fewer stated they were open to receiving additional educational messages (30.1%) (Table 2).

**Table 2:** Study participant perceptions regarding approaches to enhancing cervical cancer screening uptake

Treatment arm	Perception statements	n (%)
Intervention Group 1 SMS prompts (n = 55)		
	SMS is culturally appropriate	45 (81.82%)
	SMS informed them of the action to take	34 (61.82%)
	SMS contained useful information	29 (52.73%)
	The number of messages was adequate	27 (49.09%)
	Do not mind receiving additional cervical cancer educational messages by SMS.	17 (30.91%)
Intervention Group 2 Self-sample with HCW led education (n = 45)		
	Evalyn sterile brush was easy to use	43 (78.18%)
	I felt comfortable using the Evalyn sterile brush.	26 (57.78%)
	I experienced some pain / discomfort when using the Evalyn brush.	17 (37.78%)
	I would recommend the use of the Evalyn brush to female family.	43 (78.18%)

## Discussion

This study demonstrates the feasibility and potential acceptability of a community-based self-sampling approach for primary HPV-based cervical cancer screening, with community health workers serving as key intermediaries between the community and health facilities. HPV testing offers benefits such as better detection of high-grade cervical lesions, longer screening intervals than the pap test, and potential reductions in healthcare costs. Self-sampling further reduces provider time and associated expenses, an obvious benefit for under-resourced settings.

Importantly, only 67% of those invited to participate responded positively. Though reasons for non-participation were not documented, it is possible that barriers related to personal beliefs regarding risks, consequences and self-efficacy, presence of support from family and/or a significant other, a conducive sociodemographic circumstance and personal intention (all emphasized in models like the Theory of care seeking behaviour, The Health Behaviour Model and the Theory of Planned Behaviour) influenced decisions to opt out [24, 25].

The self-sampling approach achieved a 52.9% uptake among women with no prior screening experience, which was considered relatively acceptable. A post-sampling survey revealed that 78% found the brush easy to use and would recommend it to others. Self-sampling has shown potential to improve cervical cancer screening uptake, though its availability in rural areas remains limited due to implementation challenges [26–31]. This study highlights the potential of combining self-sampling with task-shifting to CHVs, which could improve screening coverage, especially in underserved regions aligning with WHO recommendations and furthering country efforts to meet 90-70-90 targets (vaccination, screening, treatment) as outlined in the National Cervical Cancer Elimination Plan (2026–2030) [32, 33]. Importantly, the National Cervical Cancer Elimination Plan (2026–2030) includes key interventions such as the rollout of a single-dose HPV vaccination schedule, expansion of HPV DNA testing, and the introduction of self-sampling to boost screening uptake [34]. CHVs could help extend the reach of these interventions through household visits. Importantly, about 38% of participants reported some pain or discomfort, suggesting a need for further research to compare different self-collection kits and improved training approaches.

This study also evaluated the acceptability of using health messaging to increase cervical cancer screening uptake. As mobile phone penetration in Kenya is 91%, SMS messages are seen as a cost-effective way to deliver health information [35]. Despite this, the relatively low perceived usefulness of SMS messages (52%) suggests that message framing may influence engagement. Poorly designed messages, particularly those that overlook tone, audience characteristics, or cultural context, can even have a counterproductive effect [36–38]. Additionally, health behaviour change is a complex phenomenon, that requires multifaceted interventions that go beyond awareness, to address psychological, social and contextual drivers of behaviour [25]. Nonetheless, to improve the effectiveness of future health messaging, consideration of theoretical models of health behaviour alongside collaboration with communication experts and community members in co-designing messages may enhance their impact and relevance in promoting healthy behaviors [39–42].

## Limitations

The study had several limitations. Firstly, the response rate recorded in this study is lower than response rates commonly reported in similar studies. Nonetheless, the study still provides valuable insights into intervention feasibility [43]. The requirement for mobile phone access may have influenced participant demographics, though mobile penetration in Kenya is high (91%). Future research should assess whether this affected study representativeness. Also, the imbalance in allocation across study arms substantially reduced statistical power, limiting our ability to conduct inferential analyses of cervical cancer screening uptake without risking overinterpretation of unsupported effects. Furthermore, only the self-sampling group’s responses on screening uptake were validated against hospital results. Other participants’ self-reports may reflect socially desirable answers, potentially inflating actual uptake. Additionally, the study would have benefited from data on reasons for non-participation, which would be valuable for improving future cervical cancer screening efforts. Nevertheless, findings provide important implementation insights to inform future studies and programmatic refinement.

## Data Availability

Any interested parties can apply directly to the corresponding author to access the data used in this paper by contacting them at [rosebella.ondiek@aku.edu].

## Map of Kiambu County showing the administrative units

Source: *Political Units* (Kiambu County Government), accessed from [44] https://dev.kiambu.go.ke/political-units/.

## Short message service (SMS) intervention

### Message 1 (English)

: Did you know out of every 10 cervical cancer cases, 9 are caused by persistent infection with high-risk human papillomavirus (HPV)? All women above the age of 30 years are at risk for cervical cancer. HPV testing is offered free at Kiambu Level 5 Hospital. Prioritize your health, ask for a HPV test at this hospital today.

A message from Kiambu County Community Health Services.

### Message 1 (Swahili)

: Je! Unajua kati ya kila kesi 10 za saratani ya kizazi, 9 husababishwa na maambukizo ya Human Papillomavirus (HPV)? Wanawake wote walio juu ya umri wa miaka 30 wako katika hatari ya ugonjwa wa saratani ya kizazi. Upimaji wa HPV hutolewa bila malipo katika hopsital ya Kiambu Level 5. Ipatie afya yako kipaumbele. Uliza kuhusu upimaji wa HPV katika hospitali ya Kiambu Level 5 leo.

Ujumbe kutoka Huduma za Afya ya Jamii Kaunti ya Kiambu.

### Message 2 (English)

: When detected early, cervical cancer can be treated successfully. Do not wait until it is too late. HPV testing is offered free at the Kiambu Level 5 hopsital. Ask for a HPV test at this hospital today.

A message from Kiambu County Community Health Services.

### Message 2 (Swahili)

: Inapogunduliwa mapema, saratani ya kizazi inaweza kutibiwa kwa mafanikio makubwa. Usingoje hadi kuchelewa. Upimaji wa HPV hutolewa bila malipo katika hopsital ya Kiambu Level 5. Uliza kuhusu upimaji wa HPV leo.

Ujumbe kutoka Huduma za Afya ya Jamii Kaunti ya Kiambu.

### Message 3 (English)

: A healthcare worker will insert a small brush into the birth canal to the cervix, to collect a sample of cells from around the cervix. This simple procedure is not painful. The sample is sent to the laboratory for analysis. HPV testing is offered for free at the Kiambu Level 5 hospital. Do not delay any longer, ask for a HPV test today.

A message from Kiambu County Community Health Services.

### Message 3 (Swahili)

: Daktari ataingiza brashi ndogo kwenye njia ya mtoto hadi kufikia mlango wa kizazi ili kukusanya sampuli ya seli kwenye mlango wa kizazi. Utaratibu huu ni rahisi sio chungu. Sampuli hutumwa kwa maabara kwa uchunguzi. Upimaji wa HPV hutolewa bila malipo katika hospitali ya Kiambu Level 5. Usichelewe tena. Uliza kuhusu upimaji wa HPV katika hospitali ya Kiambu Level 5 leo.

Ujumbe kutoka Huduma za Afya ya Jamii Kaunti ya Kiambu.

### Message 4 (English)

: A positive result means that an HPV infection was found, and one will be well advised of the available options for treatment. Do not miss this opportunity. Go for a free HPV test at the Kiambu Level 5 hospital today.

A message from Kiambu County Community Health Services.

### Message 4 (Swahili)

: Matokeo yakionyesha kuwa mwanamke ako na maambukizo ya HPV, atashauriwa vyema jinsi anavyoweza kupata matibabu yanaostahili. Usikose fursa hii. Nenda upate kupimwa HPV bila malipo kwenya hopsital ya Kiambu Level 5 leo.

Ujumbe kutoka Huduma za Afya ya Jamii Kaunti ya Kiambu.

## Emotions experienced by study participants who took up cervical cancer screening services during waiting period for results

**Table:**

Emotion	Usual care (n = 8) n (%)	Self-collection kits (n = 45) n (%)	Health provider (n = 11) n (%)	Total (n = 64) n (%)
Fear	5 (62.5%)	14 (31.1%)	3 (27.3%)	22 (34.4%)
Anxiety	1 (12.5%)	1 (2.2%)	0 (0.0%)	2 (3.1%)
Fear and anxiety	2 (25.0%)	16 (35.6%)	5 (45.5%)	23 (35.9%)
Calm	0 (0.0%)	14 (31.1%)	3 (27.3%)	17 (26.6%)

## Abbreviations

CHVs: Community health workers
HCWs: Healthcare workers
HE: Health education
HIV: Human immunodeficiency virus
HPV: Human papillomavirus
LMICs: Low- and middle-income countries
NACOSTI: National Commission for Science, Technology, and Innovation
NHIF: National Health Insurance Fund
OOP: Out of pocket payments
SD: Standard deviation
SMS: Short message service
